# Efficacy of a process improvement intervention on inmate awareness of HIV services: a multi-site trial

**DOI:** 10.1186/s40352-015-0023-5

**Published:** 2015-06-06

**Authors:** Holly Swan, Matthew L Hiller, Carmen E Albizu-Garcia, Michele Pich, Yvonne Patterson, Daniel J O’Connell

**Affiliations:** 1 Center for Healthcare Organization and Implementation Research, Edith Nourse Rogers Memorial VA Hospital, 200 Springs Road (152), Bedford, MA 01730 USA; 2 Department of Criminal Justice, Temple University, 1115 W. Polett Walk, Philadelphia, PA 19122 USA; 3 Center for Evaluation and Sociomedical Research (CIES), Graduate School of Public Health, Medical Sciences Campus University of Puerto Rico, San Juan, Puerto Rico; 4 Faculty Center for Excellence in Teaching and Learning, Law and Justice Studies; Rowan University, Glassboro, NJ 08028 USA; 5 Elms College, Chicopee, MA 01013 USA; 6 Center for Drug and Health Studies, University of Delaware, 257 East Main Street, Newark, DE 19716 USA

**Keywords:** HIV, Inmates, Criminal justice settings, Implementation, Process improvement

## Abstract

**Background:**

The prevalence of HIV among U.S. inmates is much greater than in the general population, creating public health concerns and cost issues for the criminal justice system. The HIV Services and Treatment Implementation in Corrections protocol of the NIDA funded Criminal Justice Drug Abuse Treatment Studies tested the efficacy of an organizational process improvement strategy on improving HIV services in correctional facilities.

**Methods:**

For this paper, we analyzed efficacy of this strategy on improving inmate awareness and perceptions of HIV services. The study used a multi-site (n = 28) clustered randomized trial approach. Facilities randomized to the experimental condition used a coach-driven local change team approach to improve HIV services at their facility. Facilities in the control condition were given a directive to improve HIV services on their own. Surveys about awareness and perceptions of HIV services were administered anonymously to inmates who were incarcerated in study facilities at baseline (n = 1253) and follow-up (n = 1048). A series of one-way ANOVAs were run to test whether there were differences between inmates in the experimental and control facilities at baseline and follow-up.

**Results:**

Differences were observed at baseline, with the experimental group having significantly lower scores than the control group on key variables. But, at post-test, following the intervention, these differences were no longer significant.

**Conclusions:**

Taken in context of the findings from the main study, these results suggest that the change team approach to improving HIV services in correctional facilities is efficacious for improving inmates’ awareness and perceptions of HIV services.

**Electronic supplementary material:**

The online version of this article (doi:10.1186/s40352-015-0023-5) contains supplementary material, which is available to authorized users.

## Background

Persons living in prisons or under criminal justice supervision in the community are at high risk for acquiring HIV, and the prevalence of HIV in criminal justice settings is substantially higher than that of the general population (Maruschak 2012). In response, the Centers for Disease Control and Prevention (CDC) has published guidelines to assist the criminal justice sector in the adoption of evidence-based practices to reduce inmate HIV risks and to provide HIV treatment in correctional settings (CDC 2009). Based on these guidelines, O’Connell et al. (2013) designed a continuum of care model for HIV services in correctional settings that includes HIV testing, prevention and education, and linkage to treatment in the facility as well as upon reentry to the community.

According to the continuum of care model for HIV in correctional settings (O’Connell et al. 2013), all persons are tested for HIV at intake to a correctional facility unless they specifically decline (i.e., opt-out testing). If the result of the test is negative, additional intensive services are not necessary through most of that person’s incarceration. If an inmate is known to be HIV-positive, or tests positive for HIV at intake, appropriate treatment, including antiretroviral medication, begins immediately and continues through their incarceration. Prior to release and community reentry, all inmates regardless of HIV serostatus, receive an evidence-based HIV prevention intervention to encourage risk reduction during the reentry transition. Examples of these may be found on the Center for Disease Control’s website (CDC Compendium of Effective Behavioral Interventions 2015) and/or on the National Registry of Effective Practices and Programs (2015). These individuals also receive comprehensive discharge planning to promote linkage to continued HIV care in the community to which they are returning. If all of these services are implemented according to this model, the evidence suggests that individual and public health outcomes would improve for this population (see O'Connell et al. 2013 for a thorough discussion of the evidence for these practices).

Many correctional facilities across the United States now offer most (if not all) of the continuum of HIV services; in fact, studies have shown that HIV outcomes (i.e., viral load suppression and CD4 lymphocyte counts) for incarcerated populations tend to improve during incarceration (Meyer et al. 2014; Baillargeon et al. 2010; Zaller et al. 2007; Springer et al. 2004; Palepu et al. 2004). However, research has also demonstrated the existence of many gaps in services along the care continuum that have resulted in insufficient HIV care, particularly during the reentry transition back to the community (Belenko et al. 2013a; Baillargeon et al. 2010; Greifinger 2010; Springer and Altice 2005; Springer et al. 2004). Implementation science has evolved in health services research in response to such service gaps. The science has been important for identifying and understanding strategies for expediting the application of best practices in diverse populations and service sectors, as well as for diverse health conditions (Brownson et al. 2012).

In response to the growing emphasis on implementation science in health and the documented gaps in HIV services for correctional populations, the National Institute on Drug Abuse (NIDA) sponsored the Criminal Justice Drug Abuse Treatment Studies (CJ-DATS), a national collaborative of investigators on three protocols designed to test implementation and process improvement strategies for improving health services for the incarcerated population (Ducharme et al. 2013). This article presents data and analyses from the HIV Services and Treatment in Correctional Settings protocol (HIV-STIC) which was developed to test an organizational process improvement strategy for implementing and improving services along the HIV continuum of care (i.e., prevention, testing, and linkage to treatment) in correctional facilities in the United States and Puerto Rico (Belenko et al. 2013b).

Investigators at each of nine research centers participating in HIV-STIC selected one or two pairs of correctional facilities in which to implement the study. Paired facilities were matched based on several factors including state/territory and type of correctional facility (e.g., security level, prison or jail). One facility from each pair was randomly assigned to either the experimental or the control condition. Prior to randomization, selected staff from all local participating facilities engaged in a one-day training on evidence-based practices along the HIV care continuum in correctional settings (for a total of 9 trainings - one per research center). At the end of the training, all staff received a directive from an executive in their administration to make improvements to services along the HIV services continuum (i.e., prevention, testing, or linkage). Staff in facilities randomized to the control condition received follow-up instructions from the executive to work on improving the services as they normally would. Staff in facilities randomized to the experimental condition composed local change teams, facilitated by external coaching from individuals trained in the NIATx process improvement model (McCarty et al. 2007), to work on improving HIV services. (See Belenko et al. 2013b for a full description of the HIV-STIC protocol design).

**Figure 1:**
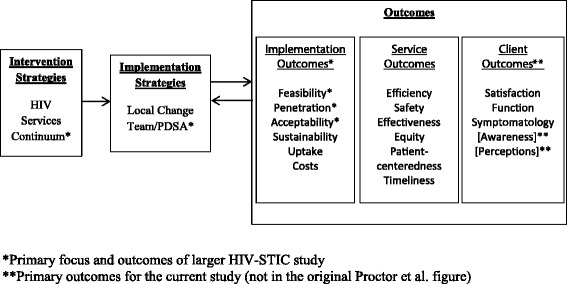
Model of Implementation Science Outcomes (Modified from Proctor et al. 2009).

The model of implementation research outcomes developed by Proctor and colleagues’ (2009) (see Figure 1) was used to frame the design of the HIV-STIC study as well as to guide the development of relevant outcome measures. The primary focus of the larger study was on implementation outcomes at the staff or organizational level of analysis (e.g., feasibility, acceptability, penetration) (see Figure 1). According to this model, achieving success at the level of implementation outcomes should trickle out to improvements in outcomes at the client level (e.g., satisfaction) in the absence of direct client interventions. As such, this paper examines the distal client outcomes of the HIV-STIC study, specifically whether the process improvement intervention involving staff was related to the awareness and perceptions of HIV services among their potential clients, that is, persons detained in the participating correctional facilities at the time of data collection. Our primary hypothesis is that compared to the Control condition, inmates who are incarcerated in facilities in the Experimental condition will express greater awareness of the HIV services continuum at follow-up. Our secondary hypothesis is that compared to the Control condition, inmates’ perceptions of and attitudes towards HIV and the provision of HIV services in facilities in the Experimental condition will be greater at follow-up.

## Methods/Design

### Data collection and measures

The Anonymous Inmate Survey was administered by locally trained research staff to groups of inmates, regardless of HIV serostatus (i.e., HIV serostatus was unknown to researchers and inmates were not recruited based on HIV serostatus), who were incarcerated at the time of data collection. Research staff worked with the correctional facility staff to schedule at date and time to administer the surveys. Facility staff then notified the inmates that they could attend the group during that time to complete a survey as part of a research study, and organized the logistics for gathering the inmates in a group. There were no inclusion or exclusion criteria aside from being incarcerated at the time of the survey administration and consenting to participate. In the group setting, the research staff informed the inmates that their participation was voluntary, that they could choose which questions to complete and/or leave the study at any time, and that not participating or only partially participating would not affect them, their treatment, or their sentence, in any way. Inmates were instructed that their answers on the survey were completely anonymous and told not to make any explicit identifying marks on the survey. Those who remained were told that by doing so, they were consenting to participate in the study. These study procedures were approved by university institutional review boards (IRB) for the research centers in the study and in many cases by an additional IRB with jurisdiction over the correctional and other agencies that were the research sites for the current study.

With the exception of two research centers, pretest surveys were administered to a convenience sample of inmates incarcerated in the experimental and control study sites within one month of randomization (i.e., pretest). Due to logistical delays in initiating the study at participating sites, staff at two research centers administered the surveys at 4 and 8 months after randomization, respectively. In both cases, surveys were administered prior to implementing the change teams. Posttest surveys were administered to another convenience sample of inmates incarcerated in the experimental and control study sites between two and six months after completion of the implementation phase of the study.

The inmate survey included 21 items focused on eliciting yes/no and Likert scale responses from each participant. A final question asked whether they would like to make any additional comments or observations, and if so, to write them in the space provided. (An additional file provides a copy of the questions asked on the survey [see Additional file 1]). To test the first hypothesis, we created an HIV services awareness index (range 0–4) by summing the responses to the four questions inquiring about participants’ awareness of the availability of any of the HIV care continuum services (i.e., HIV education and prevention, HIV testing, HIV medication, and pre-release planning services for HIV infected inmates) in the correctional facility where they were incarcerated at the time of survey administration.

To test our second hypothesis, we used three composite scales that were identified through principal axis factor analysis of twelve items asking inmates to rate the ease of using as well as how they felt about each of the HIV care continuum service components at their facility using a five point Likert scale (1–5). *Staff Impact* measured inmate perceptions of the medical, treatment, and correctional staffs’ support for HIV continuum of care on four items like “the medical staff at this institution does a good job of supporting HIV services and this institution is doing everything it can to stop the spread of HIV”. The second scale, *Medication/Pre-release Planning*, included two items: “how much [do] you believe inmates who have HIV could benefit from receiving HIV medication while incarcerated” and “how much [do] you believe inmates who have HIV could benefit from receiving pre-release planning services”*.* The final scale, HIV Education and Prevention/HIV Testing, was composed of four items related to perceptions towards these services, such as “how do you feel about HIV education and prevention classes in this institution” and “how easy would it be for you to get tested for HIV at this institution”. The internal consistency reliability for the three scales ranged from good (α = .90) to adequate (α = .68). Because we are unaware of any other surveys of inmate awareness and perceptions of the HIV Services continuum, we conducted psychometric analyses, the results of which are available from the corresponding author of this study.

Another summative composite (range 0–3) reflected whether the inmate would consider accessing HIV education and prevention and/or HIV testing. A final item asked them to rate their current level of concern about getting infected with HIV with responses ranging from 1 (Not at all) to 5 (Very Concerned).

### Analytic plan

Because it was unknown whether the surveys were completed by different inmates at each time point (due to the anonymity of respondents), a within-subjects analysis such as repeated-measures Analysis of Variance (ANOVA) could not be calculated with confidence. Instead, a series of one-way ANOVAs were first conducted to compare the experimental and control conditions on *pretest* measures of awareness, perceptions, willingness to be tested for HIV or to attend HIV prevention and education, and concern about becoming infected. A second set of one-way ANOVAs compared the experimental and control groups on *posttest* measures of the same set of variables. Cohen’s d statistic was used to calculate effect size, with .2 generally considered a small effect size, .5 a medium effect size, and .8 a large effect size.

### Sample

Seven of the nine HIV-STIC research centers collected data from 2,301 inmates for the current study: 1253 inmates for the pre-intervention and 1048 post-intervention surveys. Two research centers did not administer the Anonymous Inmate Survey due to logistical issues in obtaining permission from either the IRB or the correctional facility administrators to survey the inmates. One research center did not collect the post-intervention inmate survey due to logistical issues in obtaining permission from the correctional facility to survey the inmates. On average, each research site contributed an average of 52 (range 9–97; SD = 20.7) pretest and 58 (range 27–112; SD = 22.9) posttest surveys. The percentages of surveys contributed by each research center are presented in Table 1.

**Table 1:** **Characteristics of anonymous inmate survey sample (N = 2301)**

**Characteristic**	**Survey administration**		**% of total N**
	**% of total N at pretest**	**% of total N at posttest**	
	**(N = 1253)**	**(N = 1048)**	**(N = 2301)**
Research site			
A	7.1	8.2	7.6
B	15.2	9.5	12.6
C^1^	7.1	0.0	3.9
D	8.1	7.3	7.7
E	24.1	31.1	27.3
F	16.8	19.1	17.8
G	21.6	24.8	23.1
Sex			
Male	82.4	82.3	82.3
Female	11.1	11.8	11.4
Facility type			
Jail	31.9	34.5	33.1
Prison	68.1	65.5	66.9
State HIV prevalence category			
Low	59.9	64.1	61.8
High	40.1	35.9	38.2
Study condition			
Experimental	52.2	59.0	55.3
Control	47.8	41.0	44.7

^1^Research Center ‘C’ did not collect the post-intervention inmate survey due to logistical issues in obtaining permission from the correctional facility to survey the inmates.

As shown in Table 1, demographically, 11 percent of the inmates were incarcerated in women’s facilities. More than two-thirds (66.9%) of the inmates were incarcerated in a prison and about half (55.3%) were in correctional programs assigned to the experimental study condition. Four of the institutions housed only Spanish-speaking inmates. The Anonymous Inmate Survey was translated into Spanish for these sites. The prevalence of HIV in the state’s correctional system was taken from the Bureau of Justice Statistics report summarizing HIV/AIDS prevalence in state correctional facilities from 2008 until 2012 (Maruschak 2012). General state prevalence estimates were from taken the website of National Center for HIV/AIDS, Viral Hepatitis, STD, and TB Prevention of the Centers for Disease Control (CDC) (2015). Reflecting both the prevalence of HIV in correctional facilities as well as the CDC rank of the HIV prevalence in the states, thirty-eight percent of surveys were collected in “high prevalence” areas.

## Results

### Pretest and posttest comparisons

One-way ANOVAs on pre-intervention (pretest) awareness and perceptions of HIV services showed that inmates who were incarcerated in control facilities scored significantly higher on the HIV services awareness index, with a medium effect size [F(1, 1250) = 28.43; p = .000; Cohen’s d = .30], and on their impressions of staff impact with a small effect size [F(1, 1235) = 12.50; p = .000, Cohen’s d = .20], than inmates in facilities in the experimental condition. Conversely, the experimental group had significantly higher scores on the index of whether they would consider using HIV prevention and testing services, though the effect size was small [F(1, 1244) = 4.66; p = .031; Cohen’s d = .12] (see Table 2). The groups were not significantly different on the other dependent variables at pretest.

**Table 2:** **Comparison of dependent variable means by study condition at pre- and post-test**

	**Pretest**			**Posttest**		
**Dependent variables**	**Experimental group**	**Control group**	**p**	**Experimental group**	**Control group**	**p**
	**(n = 653)**	**(n = 599)**		**(n = 618)**	**(n = 430)**	
# of HIV Continuum Services Aware of	1.23 (1.2)	1.60 (1.3)	.000	1.39 (1.2)	1.50 (1.3)	.137
Staff Impact	2.68 (1.2)	2.91 (1.1)	.000	2.83 (1.2)	2.90 (1.1)	.361
HIV Medication and Pre-Release Planning	4.22 (1.1)	4.13 (1.1)	.142	4.11 (1.2)	4.19 (1.0)	.255
HIV Testing, Education, and Prevention	3.55 (1.0)	3.63 (0.9)	.197	3.62 (1.0)	3.65 (1.0)	.608
Consider going to HIV Education or getting tested for HIV	2.00 (1.0)	1.88 (0.9)	.031	1.90 (0.9)	1.92 (1.0)	.756
Concerned about getting HIV Infection	3.81 (1.5)	3.82 (1.5)	.918	3.87 (1.5)	3.58 (1.5)	.002

Note. The mean values of groups on dependent variables are presented in the columns, with the standard deviations appearing in the parentheses immediately below the corresponding mean. Bonferroni adjustment to correct for an inflated Type 1 error rate (i.e., .05÷12 comparisons) yielded a significance level of p = .004. Therefore, interpretation of mean differences with associated significance levels above this are at a significantly increased risk for Type 1 error.

One-way ANOVAs on post-intervention (posttest) awareness and perceptions of HIV services showed only one statistically significant difference: the inmates who were incarcerated in experimental facilities were significantly more concerned about contracting HIV, though the effect size for this difference was small [F(1, 1032) = 9.30; p = .002; Cohen’s d = .19]. No other comparisons were statistically significant.^a^

## Discussion

The comparison of the one-way ANOVA findings for pretest and posttest awareness of HIV services revealed that the differences observed on the pretest (i.e., the control group showed a greater awareness of HIV services and more positive evaluations of staff impact; the experimental group reported being more likely to consider using HIV services) did not exist at posttest. Although these findings do not reflect a direct change since the groups of inmates who were surveyed at posttest likely were not the same inmates who filled out the surveys at pretest, they do suggest that the change team intervention helped close the significant gaps identified between experimental and control groups on these key variables. Specifically, the awareness of HIV services was higher in the experimental condition at posttest than it was a pretest, and the awareness was no longer significantly lower than the control group, thus providing some support for our first hypothesis. The same holds true for the findings on staff impact, lending some support for our second hypothesis.

Collectively, these findings suggest facilities at least an indirect effect of the change team intervention on improvement in HIV services along the care continuum since inmates in the experimental condition at posttest “closed-the-gap” on differences observed on pretest awareness and perceptions of staff impact, with differences on these scores no longer significant at posttest. Finally, concern about becoming infected with HIV among inmates incarcerated in facilities in the experimental facilities at posttest was significantly higher than among inmates in the control facilities, which also suggests a distal effect of the HIV-STIC intervention to improve the continuum of HIV services.

Although these results only lend indirect support for the effectiveness of the local change team model, they should be interpreted within the context of the larger HIV-STIC study findings and per the model of implementation outcomes that framed this study and analysis (see Figure 1). Pearson and colleagues (2014) report the finding from the larger study that the local change team approach significantly improved the actual delivery of HIV services to incarcerated individuals. Our finding that the distance between the baseline differences in control and experimental samples in inmate awareness of HIV services is consistent with this implementation outcome of the larger study. The gap between the control and experimental groups’ perceptions of staff impact at baseline also narrows in the current analysis. This finding supports another main study outcome, published by Visher et al. (2014), which showed improvements in staff perception of the feasibility and acceptability of HIV services as a result of the local change team process.

### Limitations

In spite of the study’s large multi-site sample and its focus on an understudied area of HIV services research, several limitations must be considered when interpreting these findings. First, sample design was not random and the results may not be generalizable to the population in prison at the time of each data collection period. Second, HIV serostatus or risk for infection of those who chose to participate was unknown and it is possible that inmates with a greater concern about the need for HIV services were more likely to answer the study questions. We were also unable to assess study respondents longitudinally. The anonymous survey precluded identifying the respondent at baseline to enable administering the post-test measure. Future implementation research studies should explore whether these distal outcomes are replicated using repeated measures in representative samples of the population. Studies should also explore factors that could modify these findings, such as whether the facilities housed males or females and whether the facility was a jail or prison, analysis that could not be undertaken in this study due to sample size limitations.

Another limitation in the current study was response factors that were beyond the control of the researchers in terms of survey administration at sites. Two of the research centers with correctional facilities in the study did not administer the anonymous inmate survey questionnaire to inmates in their facility at baseline or follow-up, and an additional research center administered it at baseline only, due to resistance from the Institutional Review Board (IRB) or the correctional facilities administration.

## Conclusion

Comparisons between experimental and control conditions on two independent survey administrations of items related to perceptions of HIV care continuum services showed that inmates in the two conditions were initially different on three variables: 1) number of HIV services they were aware of, 2) perceptions of staff impact, and 3) willingness to seek HIV prevention and education or testing services. However, at posttest, only one significant difference was observed: the experimental group reported significantly higher levels of concern about contracting HIV. Collectively, these findings suggest at least an indirect effect of the change team intervention on improvement in HIV services along the care continuum since inmates in the experimental condition at posttest “closed-the-gap” on differences observed on pretest awareness and perceptions of staff impact, with differences on these scores no longer significant at posttest. While we must be careful not to over-state the causal effect of improvement of HIV services through this model, the current findings interpreted in the context of the larger study findings lend support for effectiveness of the local change team model on the actual provision of HIV services, improved inmate awareness of HIV services while incarcerated, and improvements in staff and inmate attitudes towards HIV services in correctional settings.

## Endnote

^a^The Bonferroni procedure for correcting for type 1 error inflation indicated an adjusted alpha level of .004 should be used.

## Additional file

Additional file 1:
**HIV Work Group Anonymous Inmate Survey.**
