# Supplementary material for: Efficacy of a process improvement intervention on inmate awareness of HIV services: a multi-site trial
Source: Health Justice. 2015 Jun 6;3:11. doi: 10.1186/s40352-015-0023-5 (PMC4507816; doi:10.1186/s40352-015-0023-5)
Supplement: Additional file 1: — HIV Work Group Anonymous Inmate Survey. [file 40352_2015_23_MOESM1_ESM.pdf]

Additional File 1. HIV Work Group Anonymous Inmate Survey

Questions/Statements	Response Set
**HIV Education and Prevention**	
Have you ever attended HIV education and prevention classes at this institution?	Yes/No
Are you aware of HIV education and prevention classes at this institution?	Yes/No
Would you consider attending HIV education and prevention classes at this institution?	Yes/No
Please indicate on the scale below how easy it would be for you to attend HIV education and prevention classes here, if you chose to do so.	Not Easy (1)- Very Easy (5)
Please indicate on the scale below how you feel about HIV education and prevention classes available within his institution.	Not Easy (1)- Very Easy (5)
Please indicate on the scale below how concerned you are about getting infected with HIV	Not Concerned at All (1)- Very Concerned (5)
**HIV Testing**	
Have you ever been tested for HIV while in prison or jail?	Yes/No
Are you aware of HIV testing services available at this institution?	Yes/No
Would you consider using these services?	Yes/No
Please indicate on the scale below how easy it would be for you to get tested for HIV at this institution, if you chose to do so.	Not Easy (1)- Very Easy (5)
Please indicate on the scale below how you feel about HIV testing services available at this institution.	Very Negative (1)-Very Postivie (5)
**HIV Medication**	
Are you aware of DOC policy at this institution for providing of medications to inmates who have HIV?	Yes/No
Please indicate on the scale below how much you believe HIV+ inmates benefit from receiving HIV medications while incarcerated.	Not at All (1)-A lot (5)
**HIV Pre-Release Planning**	
Are you aware of DOC policy at this institution for providing of pre-release planning services to inmates who are HIV+?	Yes/No
Please indicate on the scale below how much you believe HIV+ inmates benefit from receiving pre-release planning services	Not at All (1)-A lot (5)
P**erceived Value Measures**	
The correctional staff at this institution does a good job of supporting HIV services, including	Disagree (1)- Strongly Agree (5)

prevention, testing, medication for HIV+ individuals, and linking them to care when they leave prison.	
The medical staff at this institution does a good job of supporting HIV services, including prevention, testing, medication for HIV+ individuals, and linking them to care when they leave prison	Disagree (1)- Strongly Agree (5)
The treatment staff at this institution does a good job of supporting HIV services, including prevention, testing, medication for HIV+ individuals, and linking them to care when they leave prison.	Disagree (1)- Strongly Agree (5)
This institution is doing everything it can to stop the spread of HIV.	Disagree (1)- Strongly Agree (5)
People who have HIV deserve what they got.	Disagree (1)- Strongly Agree (5)
